# Resource utilization and cost assessment of a proactive penicillin allergy de-labeling program for low-risk inpatients

**DOI:** 10.1186/s13223-023-00864-6

**Published:** 2024-01-22

**Authors:** Derek Lanoue, Adhora Mir, Carl van Walraven, Timothy Olynych, Caroline Nott, Derek R. MacFadden

**Affiliations:** 1 The Ottawa Hospital, Ottawa, ON Canada; 2 The Ottawa Hospital Research Institute, Ottawa, ON Canada; 3 Division of Clinical Immunology and Allergy, McGill University, 1650 Cedar Ave., H3G1A4 Montreal, QC Canada

**Keywords:** Penicillin, beta-lactam, Oral challenge, Cost-analysis, Inpatient, Adult

## Abstract

**Background:**

Resource utilization and costs can impede proactive assessment and de-labeling of penicillin allergy among inpatients.

**Methods:**

Our pilot intervention was a proactive penicillin allergy de-labeling program for new inpatients with penicillin allergy. Patients deemed appropriate for a challenge with a low-risk penicillin allergy history were administered 250 mg amoxicillin and monitored for 1 h. We performed an explorative economic evaluation using various healthcare professional wages.

**Results:**

Over two separate 2-week periods between April 2021 and March 2022, we screened 126 new inpatients with a penicillin allergy. After exclusions, 55 were appropriate for formal assessment. 19 completed the oral challenge, and 12 were directly de-labeled, resulting in a number needed to screen of 4 and a number needed to assess of 1.8 to effectively de-label one patient. The assessor’s median time in the hospital per day de-labeling was 4h08 with a range of (0h05, 6h45). A single-site annual implementation would result in 715 penicillin allergy assessments with 403 patients de-labeled assuming 20,234 annual weekday admissions and an 8.9% penicillin allergy rate. Depending on the assessor used, the annual cost of administration would be between $21,476 ($53.29 per effectively de-labeled patient) for a pharmacy technician and $61,121 ($151.67 per effectively de-labeled patient) for a Nurse Practitioner or Physician Assistant.

**Conclusion:**

A proactive approach, including a direct oral challenge for low-risk in-patients with penicillin allergy, appears safe and feasible. Similar programs could be implemented at other institutions across Canada to increase access to allergy assessment.

**Supplementary Information:**

The online version contains supplementary material available at 10.1186/s13223-023-00864-6.

## Introduction

Approximately 10% of all patients and up to 15% of hospitalized patients [1, 2] reports a penicillin allergy. However, the prevalence of type 1 hypersensitivity-mediated reactions to penicillin is thought to be 0.065% [3]. Over 95% of self-reported penicillin allergies are determined to be inaccurate after assessment [4]. Inaccurate patient-reported penicillin allergy labels affect antimicrobial prescribing decisions, increasing the use of second-line or restricted antibiotics and possibly promoting increased antimicrobial resistance [5–13]. Numerous national and international organizations have recommended proactive penicillin de-labeling models as part of effective antimicrobial stewardship [1].

Prior cost analyses of such programs vary widely depending on the considered costs (Table 1) [7, 14–29]. Penicillin skin testing (PST) followed by oral challenge (OC) in an outpatient Allergist supervised setting has been the standard of care for penicillin allergy testing [1]. Recently, direct challenge (DC) without skin testing has been a safe and effective alternative to PST in those with low-risk allergy histories [21–24]. In general, DC is less costly than intervention models including PST (Table 1). The cost of DC ranged from $19.40 to $260.36 per patient, depending on which costs were considered. Costs of DC were less in the inpatient setting compared to the outpatient setting. Costs decreased when non-physician providers were the assessors [30], and even less so when ward nurses were able to complete the assessment process [27]. This is especially important given that most patients with reported penicillin allergies are likely to be evaluated by non-specialists due to the high prevalence of reported penicillin allergies and the limited number of Allergy and Immunology specialists [31].

**Table 1:** Summary of existing cost-analyses for penicillin allergy evaluation based on intervention type and principal provider

Study	Country	Study Design	Population	Intervention Type	Principal Provider	Cost Parameters	Findings	Inflation Adjusted Findings**
Dodek (1999)	Canada	A decision analysis	Inpatient adult	PST + OC	Allergist	Supplies and allergy consultation	PST + DC= $123.15CAD/patient	PST + DC= $186.00
Macy (2014)	USA	Retrospective, matched cohort study	Inpatient adult	PST + OC	Registered Nurse	Supplies and nursing time	PST + DC= $131.37USD/patient	PST + DC= $184.35
Ferre-Ybarz (2015)	Spain	Retrospective descriptive analysis	Outpatient n = 100 Pedatric and adult	DC vs. complete evaluation	Allergist	Allergist visit + PST + Labs + multi-dose challenge	DC= €97.19/patient PST + DC= €149.30 /patient	DC=$162.09 PST + DC= $249.00
King (2016)	USA	Retrospective analysis	Inpatient n = 50 Age ≥ 18 years	PST + OC	Allergist	PST materials	Materials alone= $96.80USD/patient	Material= $131.88
Chen. (2018)	USA	Single-center, quasi-experimentall study	Inpatient n = 58 Age ≥ 18 years	PST + OC	Allergy-trained pharmacist	Supplies and pharmacist time	PST + DC= $220USD/patient	PST + DC= $288.58
Blumenthal (2018)	USA	Time-driven activity based cost analysis	Outpatient n = 30	PST + OC	Allergist vs. nurse practitioner	Summative cost of personnel, consumables and space	PST + DC=$220 USD/patient if (allergist) PST + DC=$170 USD/patient (nurse practitioner)	PST + DC= $288.58 (allergist) PST + DC= $222.99 (NP led
Sobrino-Garcia (2019)	Spain	Single-center, prospective observational study	Outpatient n = 296 Age: ≥14	PST + OC	Allergist	Healthcare (service, materials) and non-healthcare (commute to clinic visits) direct costs, and	PST + DC=€95.2 Total costs including indirect costs estimated at €187.49	PST + DC= $147.72
Mustafa (2019)	USA	Randomized control trial	Outpatient n = 159 Age: ≥ 5 years	DC vs. PST + OC	Allergist	Allergy/immunology billing for PST, DC	DC=$107.32USD/patient PST + DC=$447.32USD/patient	DC=$138.78 PST + DC= $578.40
Ramsey (2020)	USA	Single-center, prospective, nonrandomized	Inpatient n = 100 Age: ≥ 18 years	DC vs. PST + OC	Infectious disease pharmacist	Supplies and pharmacist time	DC=$206.18USD/patient PST + DC=$419.63USD/patient	DC=$260.36 PST + DC= $529.90
Chua (2021)	Australia	Single-center, prospective, nonrandomized (assigned based on oral penicillin challenge guideline)	Inpatient n = 355 Age: ≥ 18 years	DC	Infectious disease physicians	Cost-analysis assuming ward staff can complete de-labeling tests within usual duties	DC=$35.18AUD/patient AUD=$15.52 if staff consent patient	DC=$32.36 ($14.27 if nusing consents patients)

*This literature is varied based on what costs are included and the base rates considered in the calculations resulting in wildly divergent costs between studies

**Inflation adjusted findings based on 2021 CAD^35^, *$1 USD = $1.25CAD*, €1 Euro = 1.50 CAD, $1AUD=$0.92 CAD (2021 rate)

**Fig. 1:**
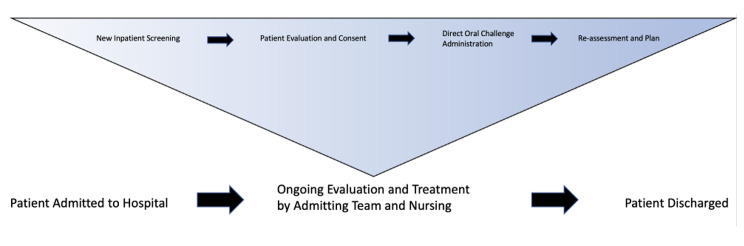
Inpatient Penicillin De-labelling Process Map

When assessing cost, Time-driven activity-based costing (TDABC) represents an accurate measurement of the rate of resource consumption for a given set of tasks [32]. To accurately measure costs using TDABC, precise data inputs are needed. For this reason, Time Motion studies, which evaluate the time spent on a given task, are often used as inputs for TDABC [33, 34]. Here, we explore the application of TDABC from the perspective of an Internal Medicine resident completing penicillin allergy assessments for inpatients. To estimate cost, we defined each step along the inpatient penicillin allergy evaluation pathway in a process map (Fig. 1). We also used the assessor’s daily mean time in the hospital to help gauge the cost of implementation for a healthcare network.

## Methods

### Study design and participants

**Fig. 2:**
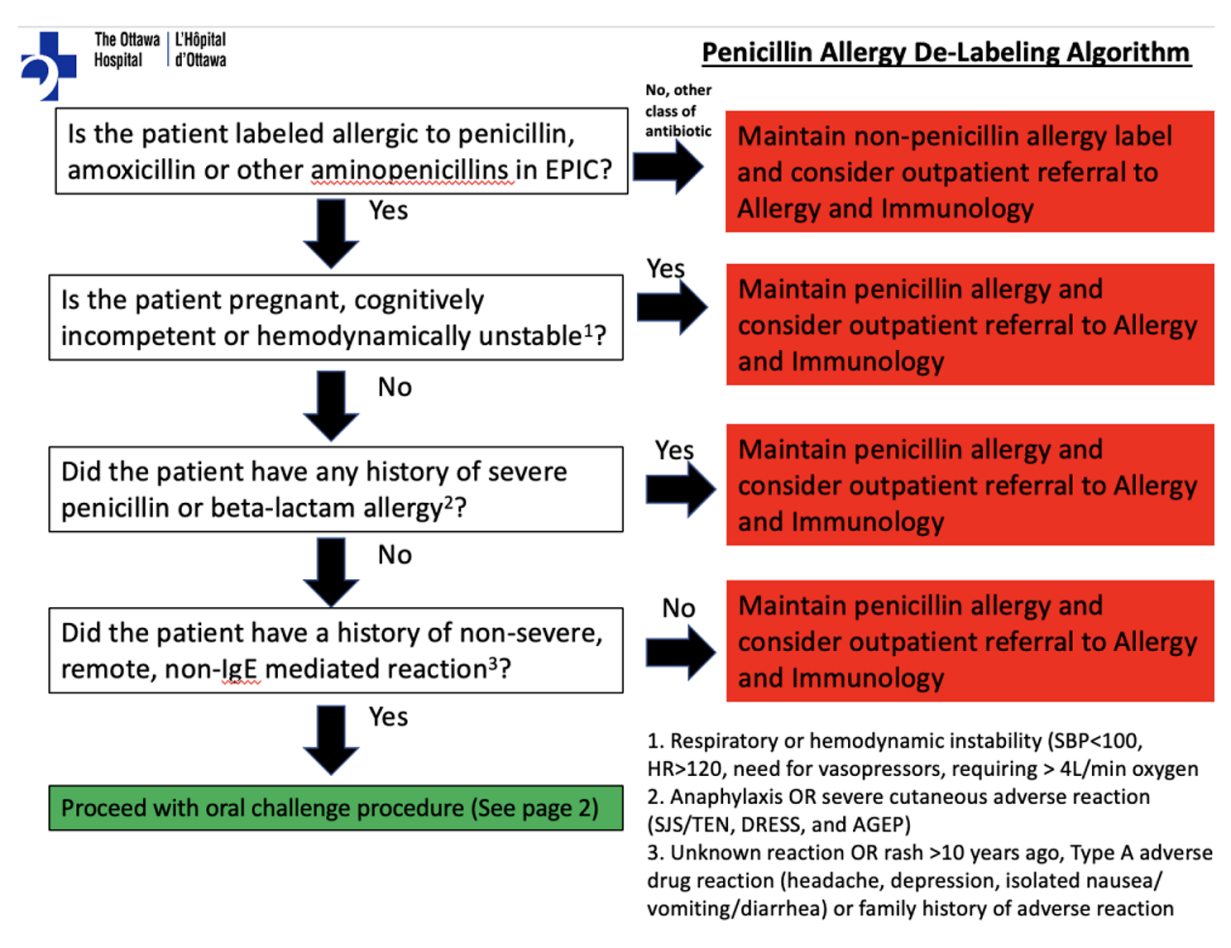
The Ottawa Hospital Penicillin Allergy Risk Stratification Algorithm

This was a single-center, prospective intervention conducted at a 1000-bed adult tertiary care academic hospital network in a single-payer healthcare system. Over two distinct 2-week periods, the assessor, a resident physician in our base case, used a daily 8am (Monday–Friday) report generator to prospectively identify and assess adult inpatients admitted to any medicine or surgical service in the previous 24 h with a penicillin allergy excluding patients with combination penicillin allergies i.e. containing beta-lactamase inhibitors. Patient records were reviewed to identify the presence of any exclusion criteria, including (a) pregnancy; (b) respiratory or hemodynamic instability (SBP < 100, HR > 120, need for vasopressors, requiring > 4 L/min oxygen); (c) admitting service psychiatry, documented history of dementia or current delirium; (d) active COVID-19 infection. The latter criterion was included for infection control reasons. The assessor approached patients without exclusion criteria after agreement from the patient’s most responsible physician (MRP). The assessor then used the Penicillin Allergy De-Labeling Algorithm approved by the Ottawa Hospital Antimicrobial Subcommittee (Fig. 2) to identify patients having a low-risk penicillin allergy history. This implementation project was designated as quality improvement by the Ottawa Hospital Research Ethics Board and thus waived from formal review.

Patients with a low-risk penicillin allergy were either directly de-labeled based on history or offered a direct oral penicillin challenge using the protocol outlined in Appendix 1. The oral challenge consisted of 250 mg PO amoxicillin and 1 h of monitoring. Vital signs must have been completed within 4 h before the challenge. Patients were educated on allergic symptoms to monitor for, including pruritus, nausea, vomiting, lightheadedness, hives, breathing difficulties or angioedema and were given a call bell to notify the assessor should symptoms develop. The assessor remained on the patient’s floor throughout the challenge for nursing reassurance. Should any symptoms develop during the challenge, the assessor would be notified at the bedside immediately. The Rapid Assessment of Critical Events (RACE) team, staffed by a critical care nurse and Intensivist, was notified of the program as an additional precaution. Following the challenge, patients were given the pager number of the penicillin allergy assessment provider should any delayed reaction develop. Patients de-labelled had their EMR updated, and a letter was sent to their home pharmacy and their family physician updating them of the completed challenge. Details of the protocol, including management of adverse drug reactions, are available in Appendix 1.

### Cost-analysis

This economic evaluation was developed in line with the Consolidated Health Economic Evaluation Reporting Standards for an economic evaluation from the perspective of the healthcare sector. All costs were inflated by the consumer price index and presented as 2021 Canadian dollars ($CAD) [35]. TDABC, a health care economic model to estimate the cost by calculating time spent on a task and the per unit cost of such task, was applied to determine the marginal cost, aka additional cost, of penicillin allergy assessment during an inpatient admission as outlined in Appendix 2. One should include the direct costs of personnel, equipment, and facilities used in patient care based on the amount of time each of these resources was used to estimate costs. In our case, similar to prior work, the specific fixed costs (hospital space, pharmacy and nursing care) were attributed to the primary reason for admission and not included.

In our base case, we calculated the labour costs using Time-Motion study methods based on intervals tracked, including screening, formal assessment, oral challenge, patient education and potential adverse drug reaction management. The analysis was based on asynchronous self-reported journaling confirmed using EHR time-stamping (Appendix 3). Data collection was accomplished through retrospective review of note creation and completion. It was pre-established that a note would be started upon initial chart review and signed following the completion of the oral challenge.

To estimate the cost of the entire assessment, we calculated the sum of the variable cost of labour, aka additional labour, needed for penicillin allergy assessment and medical consumables per patient. Given that the cost of a single dose of amoxicillin was <$0.10 CAD based on average wholesale costs, we chose not to include it in the final calculations. Yearly projections were estimated by considering the observed rate of new inpatients with penicillin allergy labels at our hospital (Appendix 4).

## Results

**Fig. 3:**
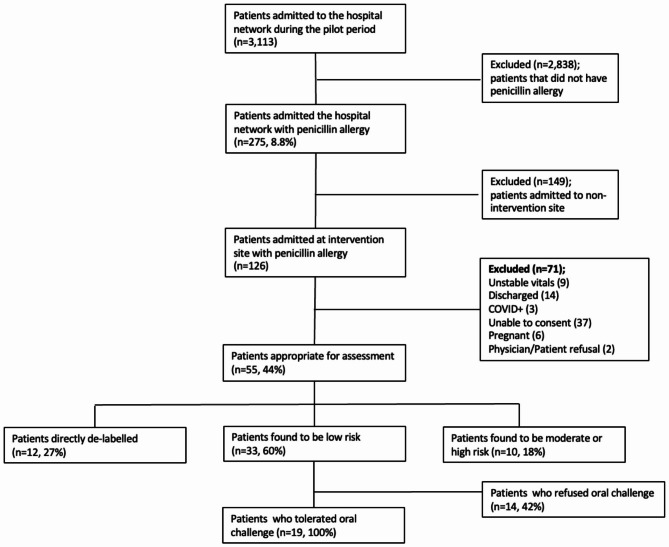
Patient Assessment Data

Our pilot intervention was implemented over two distinct 2-week periods to assess the feasibility, resource utilization, and costs of a proactive penicillin de-labeling program for low-risk inpatients. Throughout the pilot periods, we screened 1,426 admissions at the Ottawa Hospital, of which 126 patients (8.8%) were labeled as penicillin allergic based on their electronic health records. Of these 126 patients, 71 (56%) were excluded by chart review because of: COVID positivity (n = 3), persistent clinical instability (n = 9), pending discharge (n = 14), inability to provide consent (n = 37), pregnancy (n = 6), or treatment team refusal (n = 2). All remaining 55 patients had their penicillin allergy formally assessed, leading to 12 patients being directly de-labeled due to type A adverse drug reactions (family history of allergy, headache, GI symptoms, no prior administration), 10 people were classified as having a moderate or high-risk penicillin allergy; and 33 people were classified as low-risk of concerning penicillin allergy (Fig. 3).

The 33 low-risk patients were offered a single-dose supervised oral challenge; 14 declined the oral challenge and 19 accepted. All 19 patients tolerated the oral challenge without immediate or delayed adverse reactions. This resulted in a number needed to screen of 4 and number needed to assess of 1.8 to effectively de-label 1 inpatient. A total of 31/125 (24.6%) patients screened had their penicillin allergy removed.

By incorporating the single dose challenge into nursing workflow and using pre-established criteria for challenge, we sought to streamline the assessment process for low-risk inpatients. Evaluations and challenges were completed by a PGY2 Internal Medicine resident physician and were considered the base case for cost analysis. During period 1, task-specific timing was reported yielding initial daily screening of newly admitted patients of 0h04, formal assessment and consent 0h15, and an oral challenge 1h05 (Appendix 3). During period 2, the total daily time in hospital was monitored to determine the expected number of hours that would be required if an external assessor was to be hired. The time in hospital began with initiation of patient screening and ended after the final patient’s observation finished and patient education was complete. The results yielded a median time in hospital of 4h08 (0h05, 6h45).

Annual administration of such a program would result in approximately 715 penicillin allergy assessments and 403 patients de-labeled assuming 20,234 annual weekday admissions and an 8.9% penicillin allergy rate. Applying a one-way sensitivity analysis to the assessor used (Table 2), the annual cost of administration (Monday–Friday) would be between $21,476 ($53.29/patient de-labelled) for a pharmacy technician and $61,121 ($151.67/patient de-labelled) for a Nurse Practitioner or Physician Assistant after appropriate training.

**Table 2:** Summary of cost-analyses for penicillin allergy evaluation based on type of assessor

Penicillin Allergy Assessment Provider	Hourly Wage ***	Cost per Assessment Day (4.13 h)	Annual Program Cost (52 weeks, 260 days)	Cost per Penicillin Allergy Assessment (715)	Cost per Patient De-Labelled (403)
Internal Medicine Resident	$32.20	$133.00	$34,580	$48.36	$85.81
Physician Assistant	$56.92	$235.08	$61,121	$85.48	$151.67
Nurse Practionner	$56.92	$235.08	$61,121	$85.48	$151.67
Registered Nurse	$38.92	$160.74	$41,792	$58.45	$103.70
Pharmacist	$50.82	$209.93	$54,582	$76.34	$135.44
Pharmacy Tech	$20.00	$82.60	$21,476	$30.04	$53.29

***Wages calculated based on median hourly wage in Ontario, Canada in 2021 using statistics Canada labour force data [31]

## Discussion

When we compare the various methods of penicillin allergy assessment, as outlined in Table 1, the costs vary widely depending on several factors. Such factors include the type of practitioner performing assessments, skin testing vs. direct challenge, inpatient vs. outpatient and costs attributed to the de-labelling process. Our study used a decision tool similar to prior published works that pre-select for a low-risk patient population. This minimizes the risk of adverse events and limits the number of daily challenges. Despite our restrictive screening criteria, we de-labelled 24.6% of new inpatients with penicillin allergy, a rate similar to previously reported inpatient de-labeling work by Ramsey et al. who de-labeled 24% of patients screened using a combination of skin testing and direct challenge [22]. We presented the cost of various healthcare practitioners who could be capable or have been previously demonstrated to be capable of leading a similar initiative. Our main cost analysis input, the daily median time in the hospital, allowed for the most realistic estimate of resource utilization given allied healthcare providers are typically paid hourly rather than by task and significant assessor downtime was observed. The lowest costs observed in the literature were in Australia for inpatient direct oral challenges at $14.27 per assessment [27]. However, in this study, Chua et al. assumed all costs related to the challenge, including assessment and patient consent, are usual nursing duties and are not included in the cost of de-labeling. Although ideal, this does not occur at our institution. For this reason, we sought to determine the impact of a dedicated provider to facilitate these tasks. The most expensive de-labeling occurred in the US and was reported by Mustafa et al. at the cost of $578.40[23], which was an outpatient allergist-led assessment which included skin testing and oral challenge. These costs were largely driven by outpatient healthcare facility costs which were attributed to the primary reason for admission and as such not included in our study. A final analysis by Sobrino-Garcia et al. proposed that the total cost of outpatient assessments may be as much as 100% higher [25] than direct healthcare costs when non-healthcare-related expenditures are considered, such as patient transportation and opportunity costs. Avoiding these indirect costs is another potential benefit of inpatient de-labeling. Furthermore, hospitalized patients are often sicker than outpatients benefiting more urgently from antibiotic changes. They may have even more challenges attending what can be multiple visits required for an outpatient drug allergy assessment.

Key limitations of our study include the small sample size of patients, in which an adverse drug reaction was not observed. Management of adverse events may increase the time spent by penicillin allergy assessment providers and therefore increase the cost. Given the expected rate of penicillin allergy adverse reactions in the pre-selected low-risk population has been described as being 2% [22], a substantially large sample size would be required to adequately evaluate costs associated with managing adverse outcomes. Furthermore, evaluation times would be longer and potentially costlier if patients requiring substitute decision-makers were included in the study. Patients with documented dementia, delirium or patients admitted to a psychiatric unit were excluded due to concerns regarding reliable allergy history. We used a resident physician as our base case; the various healthcare providers’ efficiency and time to perform these tasks may differ from that of the resident physician and need further validation. The time-motion study, which formed the basis of input data, was limited by being from a single resident physician provider. Time motion data was largely based on asynchronous self-reporting which is prone to errors in temporal perception and memory. The speed of assessments could have also been affected by the act of measurement. However, the majority of tasks were verified by timestamp records on patient EMR and in alignment with previously reported data. The interpretation of time intervals must take into consideration the residents’ experience and comfort level with the use of the electronic health record system, penicillin allergy assessment and administration of oral challenges. Should the study be replicated with a less experienced provider, preceding training and the associated costs would be included in total cost of implementation. Efficiency of assessment was also significantly improved through the use of assessment templates and order sets. The time to develop such templates was also not considered in the time-motion study.

Future directions should include a more targeted approach to patients who may benefit most and weekly rather than daily assessments to reduce the cost of administration further. Alternatively, the use of web-based guidance tools such as penicillinallergy.ca to help primary treatment teams screen and challenge patients independently. If one was to include patients with cognitive impairment, one could consider using substitute decision-makers with good knowledge of patient’s medical history and performing skin testing to assess these patients. Further research is needed to develop an approach to evaluate patients with known cognitive impairment safely.

In conclusion, our analysis of a non-allergist-led inpatient penicillin allergy de-labeling program adds to a growing collection of studies outlining the feasibility, cost and expected benefit of such programs. These examples can serve as blueprints for institutions wishing to implement inpatient penicillin allergy assessment programs as part of their antimicrobial stewardship initiatives. The tasks performed by the resident physician in this study may be extended to allied health professionals such as nurses, pharmacists or physician assistants with relevant training, further reducing costs and increasing accessibility.

### Electronic supplementary material

Below is the link to the electronic supplementary material.

Supplementary Material 1

## Data Availability

All data generated or analyzed during this study are included in this published article.

## Abbreviations

DC: Direct oral challenge
PST: penicillin skin testing
OC: Oral challenge
MRSA: methicillin-resistant Staphylococcus aureus
VRE: vancomycin-resistant Enterococcus
$: 2021 Canadian dollars
