# Supplementary material for: Resource utilization and cost assessment of a proactive penicillin allergy de-labeling program for low-risk inpatients
Source: Allergy Asthma Clin Immunol. 2024 Jan 22;20:7. doi: 10.1186/s13223-023-00864-6 (PMC10804656; doi:10.1186/s13223-023-00864-6)
Supplement: Supplementary file 1 — Supplementary Material 1 [file 13223_2023_864_MOESM1_ESM.docx]

**Appendix 1: Oral Challenge Algorithm**

**
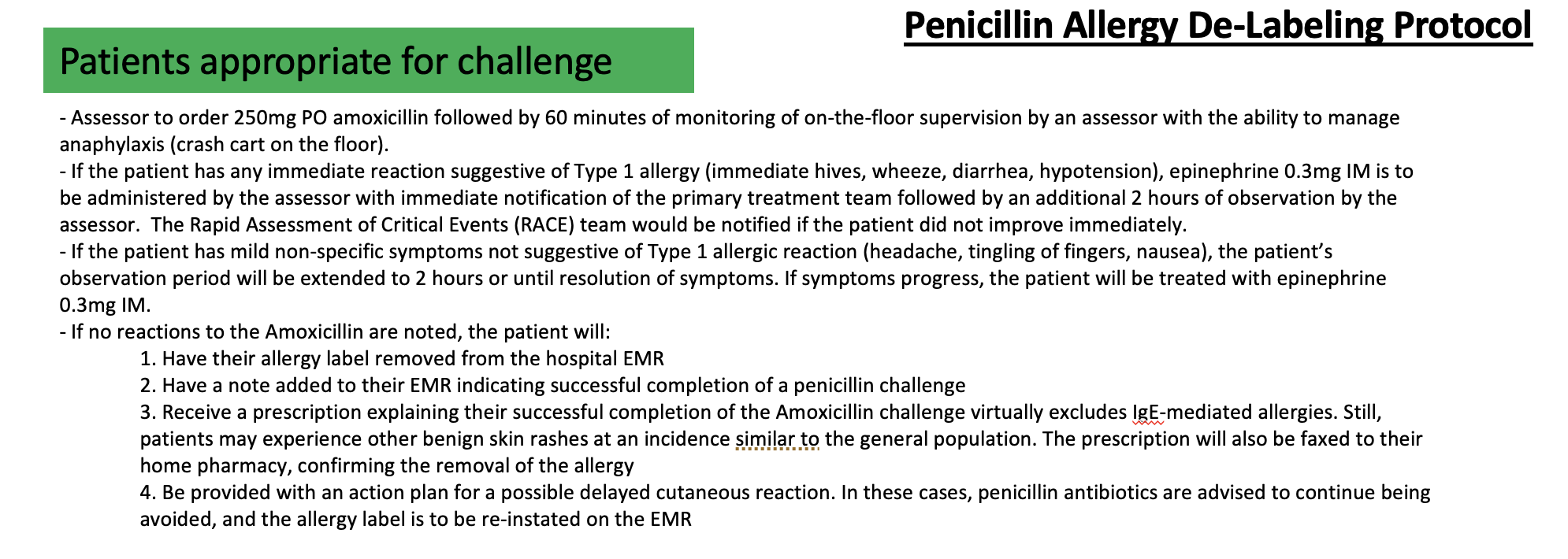
**

**Appendix 2: Process Map**


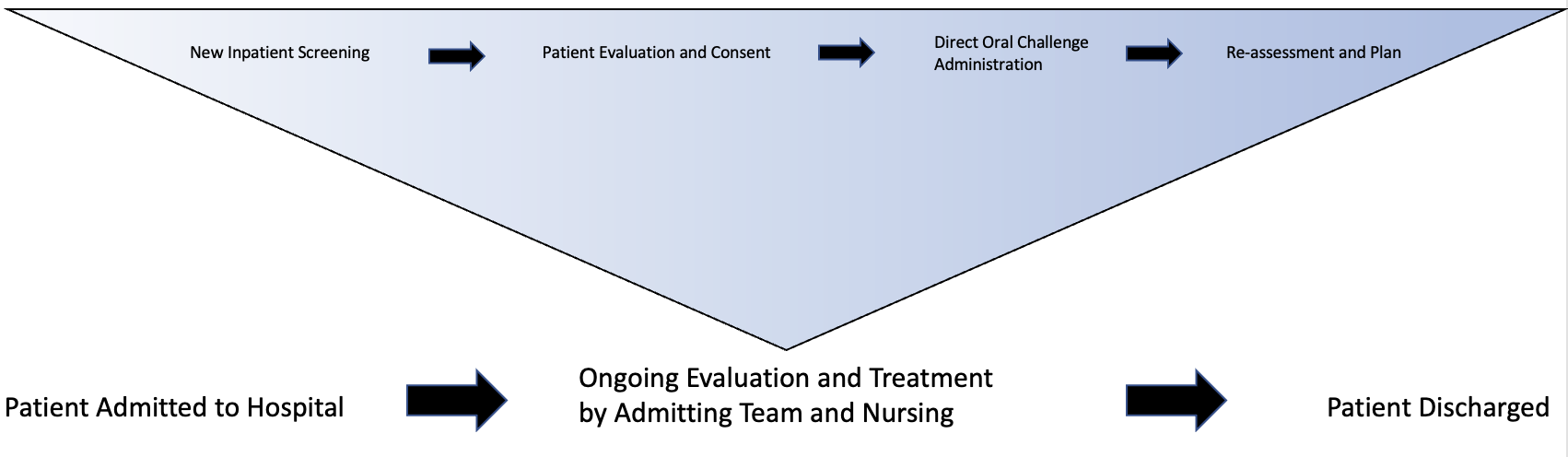


**Appendix 3: Study parameters**

Period 1:

Screening of newly admitted patients=4 minutes

Formal patient assessment=15 minutes

Oral challenge and patient education= 65 minutes

Period 2: Daily assessment times

Mean = 3:46 (0:05, 6:45)

Median = 4:08

| Date | Total Time in Hospital |
| --- | --- |
| February 15th, 2022 | 6:35 |
| February 16th, 2022 | 5:37 |
| February 17th, 2022 | 3:40 |
| February 18th, 2022 | 4:15 |
| February 22nd, 2022 | 0:05 |
| February 23rd, 2022 | 0:15 |
| February 24th, 2022 | 5:45 |
| February 25th, 2022 | 4:01 |
| March 7th, 2022 | 3:30 |
| March 8th, 2022 | 4:32 |

**Appendix 4:Cost Analysis**

Resident physician-led assessments (base wage $32.20/h CAD)

-Using a median time in hospital of 4 hours and 8 minutes and an hourly rate of $32.2/h the administration costs would equate to $133 per day (4.13x32.2).

-There would also be an average of $0.09 per day in material costs of amoxicillin.

-As such, if the program were to be administered Monday to Friday for an entire year (52 weeks) would cost a total of $34,580 beyond the fixed hospitalization costs of the patients.

-During our 4 week intervention, 55 patients had their penicillin allergy formally assessed leading to 31 patients being de-labeled. This resulted in a number needed to screen of 4 and number needed to assess of 1.8 to remove one inappropriate penicillin allergy label.

-A single site at our institution based on the observed data would result in 20,234 yearly weekday admissions. Assuming the 8.9% penicillin allergy rate we observed there would be 1800 patients screened resulting in approximately 715 penicillin allergy assessments and 403 patients de-labelled.

-As such, the average cost per penicillin allergy assessment would be $48.36 including challenges if appropriate. ($34,580/715=$48.36)

The average cost per patient de-labelled would be $85.81
